# Presence and Persistence of Ebola or Marburg Virus in Patients and Survivors: A Rapid Systematic Review

**DOI:** 10.1371/journal.pntd.0004475

**Published:** 2016-02-29

**Authors:** Julii Brainard, Katherine Pond, Lee Hooper, Kelly Edmunds, Paul Hunter

**Affiliations:** 1 University of East Anglia, Norwich, United Kingdom; 2 University of Surrey, Guildford, United Kingdom; Tulane School of Public Health and Tropical Medicine, UNITED STATES

## Abstract

**Background:**

The 2013–15 Ebola outbreak was unprecedented due to sustained transmission within urban environments and thousands of survivors. In 2014 the World Health Organization stated that there was insufficient evidence to give definitive guidance about which body fluids are infectious and when they pose a risk to humans. We report a rapid systematic review of published evidence on the presence of filoviruses in body fluids of infected people and survivors.

**Methods:**

Scientific articles were screened for information about filovirus in human body fluids. The aim was to find primary data that suggested high likelihood of actively infectious filovirus in human body fluids (viral RNA). Eligible infections were from Marburg virus (MARV or RAVV) and Zaire, Sudan, Taï Forest and Bundibugyo species of Ebola. Cause of infection had to be laboratory confirmed (in practice either tissue culture or RT-PCR tests), or evidenced by compatible clinical history with subsequent positivity for filovirus antibodies or inflammatory factors. Data were extracted and summarized narratively.

**Results:**

6831 unique articles were found, and after screening, 33 studies were eligible. For most body fluid types there were insufficient patients to draw strong conclusions, and prevalence of positivity was highly variable. Body fluids taken >16 days after onset were usually negative. In the six studies that used both assay methods RT-PCR tests for filovirus RNA gave positive results about 4 times more often than tissue culture.

**Conclusions:**

Filovirus was reported in most types of body fluid, but not in every sample from every otherwise confirmed patient. Apart from semen, most non-blood, RT-PCR positive samples are likely to be culture negative and so possibly of low infectious risk. Nevertheless, it is not apparent how relatively infectious many body fluids are during or after illness, even when culture-positive, not least because most test results come from more severe cases. Contact with blood and blood-stained body fluids remains the major risk for disease transmission because of the known high viral loads in blood.

## Author Summary

The 2013–15 Ebola outbreak has been much larger and lasted longer than any previous Ebola emergence, and is further unusual because of the thousands of survivors left behind. In 2014 the World Health Organization stated that lack of evidence made it impossible to give confident guidance about which body fluids of Ebola patients or survivors are infectious. This review summarises published evidence on presence of Ebola and the closely related Marburg virus (filoviruses) in body fluids of infected people and survivors though end July 2015. Scientific and other reports were searched for tests to detect filovirus in human body fluids on otherwise confirmed cases. 33 reports had relevant test results. Blood products were confirmed as likely to be highly infectious among actively ill cases. In actively ill patients, filovirus was often found in non-blood body fluids. However, (apart from in semen), it is rare for viable virus to be found in survivors. It is not apparent how relatively infectious most body fluids are, due to inconsistencies in testing techniques and lack of data about how much virus is present at different points of illness and recovery.

## Introduction

The 2013–15 epidemic of Ebola virus disease in West Africa was the largest recorded filovirus outbreak, as well as the first emergence of the Zaire species of Ebola in a high-density urban setting. It is generally accepted that Ebola is a zoonotic infection whose primary reservoir is probably bats [1]. Contact with wildlife generates a small number of index patients [1,2], and widespread and sustained disease transmission can follow in community settings, with a subsequent high mortality rate [3].The size of the 2013–15 outbreak increased the need for better understanding of all transmission pathways and specific types of exposure. Hence, much of the previous advice and guidelines needs to be critically reviewed, particularly with regard to risks within communities.

It is now generally accepted that both Ebola and the closely related Marburg virus diseases, are typically transmitted through direct or indirect contact with body fluids of an infected individual [4]. However uncertainties remain about which body fluids are infectious and when they pose a risk [5]. In order to address these concerns the World Health Organization released a set of “Q and As” on sanitation concerns during the 2013–15 outbreak [6], which also stated that there was insufficient evidence to give definitive guidance. In order to undertake an adequate risk assessment, knowledge on the presence of the virus in various body fluids is essential. We report a rapid systematic review of the available published evidence on the presence of filoviruses in body fluids of infected people and survivors.

## Methods

### Searches

Medline, Scopus and grey literature (S1 Table) were searched through 23 July 2015 with the string filovir*.*af*. *OR ebola*.*af*. OR ebolavir*.*af OR Marburg-virus*.*af*, without restrictions for date or language.

### Inclusion criteria

The aim was to find primary data that suggested high likelihood of actively infectious virus in human body fluids (viral RNA). Eligible infections were from any of the Marburg virus species and Zaire, Sudan, Taï Forest and Bundibugyo species of Ebola. Species of filovirus not known to be dangerous to humans were excluded. Cause of infection had to be laboratory confirmed (culture or polymerase chain reaction/RT-PCR tests), or evidenced by compatible clinical history with subsequent positivity for filovirus antibodies or inflammatory factors. Post-disease markers in body fluids were not deemed eligible by themselves to confirm cause of disease, because antibodies are widespread in the regional population, including in many people with no relevant clinical history [7–11].

Commentaries, editorials, news reports, protocols or conference presentations were excluded. We took it as given that any patient ill with filovirus should have detectable virus in blood. Therefore, studies which only report a single test result on blood for each patient or did not indicate the patient(s)’ day of illness were ineligible: they did not add to our objectives of describing viral load change over time in blood, or the likelihood of virus in non-blood products. Similarly, articles which reported primary data that were duplicated elsewhere were not included. Titles and abstracts were screened for inclusion criteria by a single reviewer and verified by a second reviewer. Where abstracts were unavailable, the paper was only assessed in full text if the title included at least one of these keywords: *survive*, *survival*, *fluids*, *viral load*, *urine*, *semen*, *saliva*, *plasma or blood*. Inclusion of full text papers was assessed in duplicate and decision differences at all stages were resolved by discussion. Articles were grouped where they reported on the same primary data, to ensure patient results were not duplicated. We searched selected papers for further articles relevant to our research questions.

Data were extracted from included studies by a single researcher and verified by a second researcher, for patients with confirmed filovirus infection. Details extracted were: bibliographic details, virus species, date and place of outbreak, body fluids tested, number of people tested and number of samples, days following disease onset and assay methods.

A PRISMA checklist [12] is provided (S2 Table). Study validation (S3 Table) was primarily concerned with reliable confirmation of disease cause and presence of eligible filovirus RNA. Validity questions were intended to verify that assay methods were appropriate and validated, that specimens were duplicate tested or compared to controls, and that samples were handled and stored correctly for a relatively short period before testing (< 2 weeks, to reduce the risks of specimen degradation).

Data on virus concentration in blood samples is presented in graphic form. Presence or absence of virus in non-blood body fluids was presented as proportion positive with 95% confidence intervals. In order to adjust for multiple samples from the same patient, each specimen was weighted by the inverse of the number of samples from that patient: To prevent a large number of test results from a small number of patients skewing the predicted probability of positivity, each test result was weighted by the inverse of the number of samples from that patient. Thus, if ten test results were from one patient, each carried a weight of 0.1, but if only one test result existed for another patient, the latter test had a weight of 1 in constructing the probability distribution. Proportions and associated confidence intervals (on a Poisson distribution) were calculated using Stats Direct 3 and the results presented using fir tree diagrams, for samples taken in the first 16 weeks after onset of illness only.

## Results

**Fig 1:**
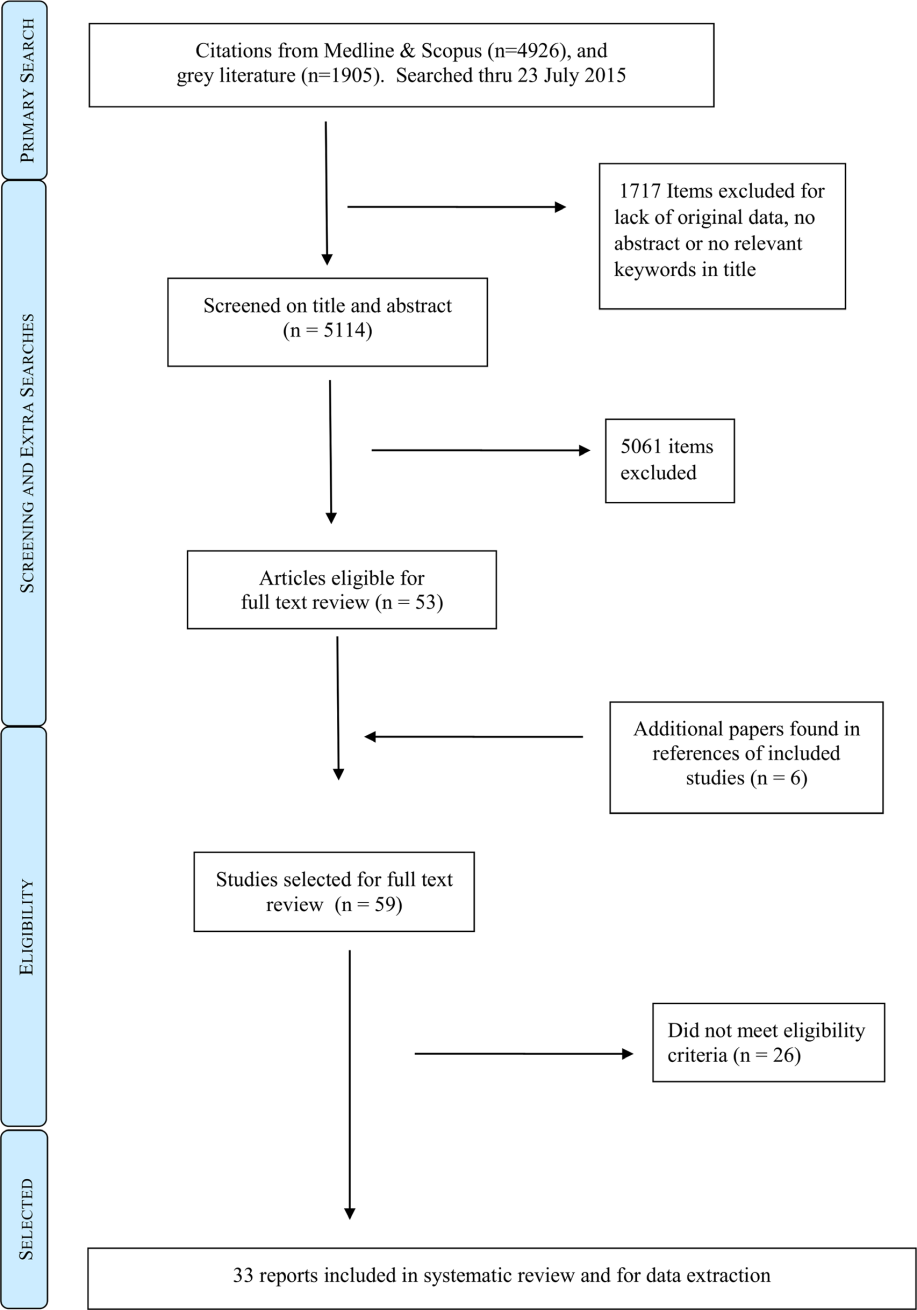
Study selection procedure.

4926 unique articles were found in Medline and Scopus (Fig 1), and 1905 items were found in grey literature. 114 entries were immediately excluded for being conference abstracts, protocols, news reports, commentaries or editorials. A further 1603 items lacked an abstract. Most of these appeared to be short commentaries, news summaries and possible conference presentations; none contained keywords relevant to our review. Thus, 5114 references were screened on title and abstract. Of these, 51 articles were chosen for full text review, of which one came from the grey literature search (S1 Table). Six articles were cited by other literature and also eligible for full text review. Twenty-six articles were excluded after full text review for not meeting the eligibility criteria. This left 33 selected articles for which data were extracted and summarised (Table 1). Detailed validity assessment is available (S3 Table).

**Table 1:** Selected study characteristics.

Species, authors	Outbreak dates and treatment place	Body fluids tested (No. patients: No. samples)	Assay method(s)
EBOV[7]	Sep-Oct 1976, Yambuku, Zaire	Blood (1:10)	Platelet counts, antibody titers
EBOV[20]	November 1976, England UK	Blood (1:21) Faeces (1:5), Urine (1:7) Seminal fluid: (1:5) Throat swab: (1:2)	Cell cultures and microscope examination
EBOV[21]	Yambio, Sudan 1979	Blood and liver samples. 189+ specimens tested: 10 cases detailed for data extraction	Vero cells, cavy tissue cultures (virus isolation), Sera antibody and isolation of virus in post-mortem tissues
EBOV[14]	Summer 1995, Kikwit DRC	Blood (50+:465+)	Vero cell cultures confirmed by antigens/antibodies
EBOV[17]	2–13 July 1995, Kikwit DRC	Blood products, conjunctival, rectal, saliva/oral, seminal, urine, vaginal, (12 pts:1–4 samples each) during convalescence	RT PCR, cell cultures and genetic sequencing
EBOV[16]	Jun-July 1995, Kikwit DRC	Blood products, tears, sweat, stools, saliva/oral, semen, urine, vaginal, (28+:300+), mostly convalescents	Virus isolation, RT-PCR, antigens
EBOV [22]	Oct-Nov 1996, Johannesberg SA	Blood plasma (1:2), semen (1:1)	Vero cell culture and unclear but repeated test methods, genetic sequencing
EBOV[23]	Early 2003, Kéllé, Cuvette Ouest, Congo	Oral and blood specimens from 24 cases & 10 controls	RT-PCR, antigens and antibodies, genetic sequencing
EBOV [24]	Jul-Aug 2014, Sierra Leone	Blood (35:38)	RT-PCR
EBOV[25]	August 2014, Sierra Leone, transfer to Germany	Blood plasma (1:12), Sweat (1:19), Urine (1:18)	RT-PCR and Vero cell cultures
EBOV [15]	Sierra Leone 2014	Blood serum (≥ 6: 266), Throat wash (> 1: 49)	RT-PCR
EBOV [26]	Sep-Oct 2014, Sierra Leone transfer to Frankfurt Germany	Blood products (1:23), Stool (1:9), Urine (1:8), other liquid waste (1:3)	RT-PCR
EBOV [18,19,27–29]	Jul-Oct 2014, 4 transfers from West Africa to USA	Blood (4:107), Urine/renal waste (1:3)	RT-PCR
EBOV [30]	Oct 2014, Spain	Blood (1:2), Conjunctival (1:5), Saliva (1:6), Sweat (1:6), Stool (1;5), Urine (1:5), Vaginal (1:5)	RT-PCR and Vero cell culture
EBOV [31]	Sep-Oct 2014, Texas USA	Blood (3:16), Rectal (1:1), Skin (1:2), Sweat (1:1), Throat (1:1), Urine (2:10), and Vaginal (2:3)	qRT-PCR
EBOV [32]	Oct 2014, Liberia transfer to USA	Blood (1:10)	RT-PCR
EBOV[33]	Oct 2014, Guinea	Urine (1:1), Blood (1:3), Breastmilk (1:1)	RT-PCR
EBOV [34]	West Africa, 2014–2015	Blood (1:4)	qRT-PCR
EBOV [35]	Oct-Nov 2014, New York City	Blood (1:3)	NAAT
EBOV [36]	Convalescent detained in India	Semen (1:2)	unclear
EBOV [37]	Nov 2014, Monrovia	Blood (2:6)	RT-PCR
EBOV [38]	Nov 2014, Sierra Leone transfer to Switzerland	Blood (1:12), Conjunctival (1:7), Saliva (1:6), Stools/Rectal (1:8), Sweat (1:3), Urine (1:11)	RT-PCR
EBOV [39]	Sept 2014 and March 2015, Liberia	Blood (1:2), Semen (1:1)	RT-PCR
EBOV [40]	Convalescent in USA, March 2015	Blood (1:2), Conjunctival (1:3), Semen (1:1), Urine (1:1)	RT-PCR
MARV[41]	Sept. 1967, Marburg, Germany	Semen (1:1)	Virus antigen in semen, wife contracting disease after sexual intercourse and cell culture test to confirm wife’s illness
MARV[42]	Sept. 1967, Marburg, Germany	Blood (17:17), Stools (5:5), Throat (6:6), Urine (4:4)	Cell cultures
MARV[43]	Feb 1975, Johannesburg South Africa	Fluid aspirated from eye (1:2)	Vero cell culture
MARV [44]	2008, Colorado	Blood (1:2)	RT-PCR and culture
SUDV[13]	2000 Gulu Uganda	Breastmilk (1:2), Saliva (10:16), Semen (1:2), Sputum (1:2), Stools (4:4), Sweat (1:1), Tears (1:1), Urine (5:11), Vomit (1–2:2)	RT-PCR and virus culture
SUDV[45]	2000–2001, Uganda	Blood products, (many pts but only six in detail)	RT-PCR (variants), antigen-capture diagnostic assay, plaque assays (Vero cell cultures)

Notes: RT-PCR = Reverse transcriptase polymerase chain reaction. NAAT = nucleic acid amplification test, MARV = Marburg virus, SUDV = Sudan Ebola, EBOV = Zaire Ebola.

The total number of patients who provided which types of samples in which time period cannot be calculated precisely because of imprecise day-of-illness information reported in some papers [13–15]. There is also the potential problem that some samples were provided by the same patients in different articles (eg [16] and [17], or [18] and [19]). We corrected for this where known and appropriate (to avoid duplicated data being reported twice), but there is a small chance that a few patients were double-counted in our totals. Nevertheless, some overview comments about data availability can be made with confidence.

Apart from saliva and blood, there are potentially important gaps in the information on all bodily fluids. There were too few samples to allow strong conclusions to be made for breastmilk, vomit or sputum (fewer than 4 patients gave samples, with virus not detected in all, even during active illness). There were no semen samples before day 32 of illness, and just 17 patients in total provided all semen samples (positivity in first 7 months after disease onset = 70%). The patient numbers for eye fluids, skin, sweat or vaginal swabs samples before day 17 of illness was just 4–9 individuals. In contrast, at least 14 patients provided samples for each of stools, urine, saliva or blood products by the 16^th^ day of illness. In the period through 112 days after onset of illness, between 15 and 45 patients gave eye, skin, sweat and vaginal specimens Saliva and blood drew on samples from 90+ unique patients of which at least 70 samples (50+ patients, saliva) and > 200 samples (145+ patients, blood) were collected before day 16. However, after day 16, blood samples were provided by just twelve of the uniquely identifiable 145 patients.

Body fluid data were found for Marburg (MARV) but not Ravn virus, and for Ebola species EBOV and SUDV but not TAFV or BUDV. Presence of actively infectious virus in body fluids was reported in six different ways. Precise viral load was not usually reported, and there were problems with the consistency and accuracy of the assay tests used. Hence, virus presence in blood is reported in our summary according to assay method (culture, RT-PCR or NAAT) and units given by authors, but with many caveats, and in non-blood body fluids are mostly reported as simply positive or negative.

### Blood or blood products

Twenty-nine studies reported on filovirus detection in blood either in a time series for individual patients or for a group of five or more patients. 26 articles were concerned with Zaire Ebola, two with Marburg virus [42] and one with SUDV Ebola [45]. Many reports [n = 14; 17,21–23,27,30,31,33,35–37,40,42,44] merely reported presence or absence of viral RNA. Six papers gave viral load/concentration in blood as CT values [19,24,28,32,34,39] and two articles [7,18] stated tissue-culture infectious dose (TCID_50_). A 1978 paper reported infectious units [20] and five studies gave results in copies per ml [15,25,26,38,45]. Plaque-forming units (PFU) were also reported [14]. There was also a mix of detection methods (RT-PCR, culture and NAAT), and some papers gave results for duplicated samples by two different assay methods with different units (eg., CT values by RT-PCR and presence/absence by culture [19]). Sometimes for reporting purposes, authors converted their observed metrics (such as CT values) to another reporting unit (eg., TCID_50_, [18]).

Standardising these results to a single measurement unit was problematic. Multiplying TCID_50_ by 0.7 for adults converts to PFU [46] and conversion curves from CT values to TCID_50_ have been produced for Ebola virus [18], but translating a mere presence/absence test to other units is less straightforward. Viral loads were often presented in imprecise graphic forms [bar charts or scatterplots, 14,25,28,32,38,45]. Confidence intervals or ranges for viral load estimates were provided (graphically) in only one paper [14]. As a result, most viral loads were somewhat inexact, lacked confidence intervals and are not equivalent between patient groups.

**Fig 2:**
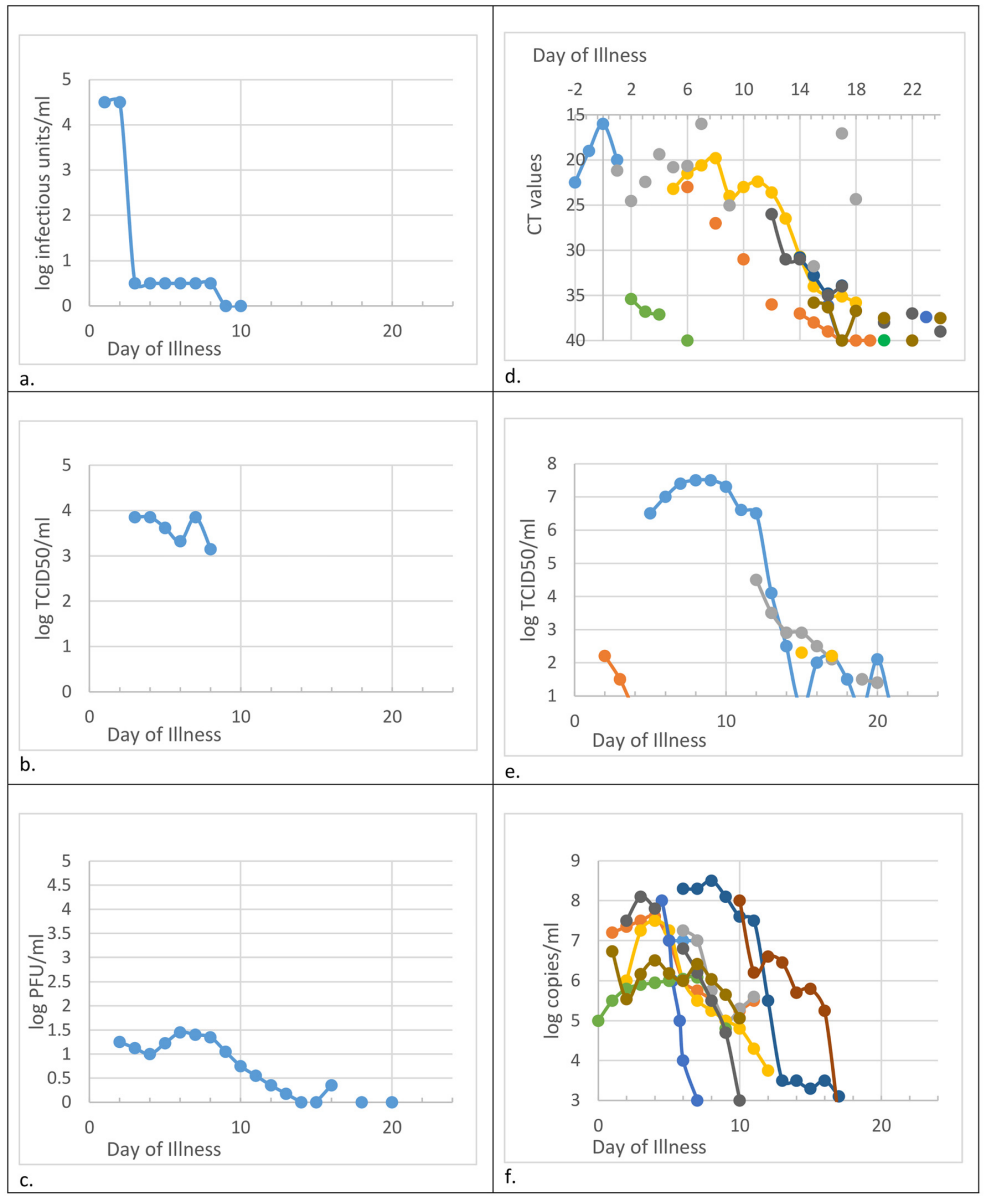
Viral load from blood samples in filovirus patients. Results are for blood or blood products (serum or plasma) until day 24 of illness. Units are as stated in cited articles. Leftside panels a-c: culture only detection methods. Right-side panels d-f: RT-PCR detection only. Bottom chart value = stated limit of detection ([18,25,26,38], all data in panel d) or implied detection threshold (all other sources). *Panel source data*: a. 1 patient in [20]; b. many patients in [7]; c. averages from many patients in [14]; d. one patient from each of [32,34,39], two patients in [28], four patients in [19] (including the two patients in [28]), many patients in [24]; e. four patients in [18] who are same four patients as in [19] (part of panel d, also duplicated two patients in [28]); f. one patient from each of [25,26,38], six patients in [45] and many patients in [15].

The blood measurements should still indicate when viral loads are probably highest and when they are likely to approach undetection. Fig 2 shows viral load information in blood or blood products from culture methods (left side panels) or RT-PCR (right side), in the units reported by the stated authors, plotted against corresponding day of illness. The limit of detection (LoD) is the bottom value on each chart, either stated or implicit in the reporting methods. It merits mention that the true LoD for CT values (Panel d) may be uncertain. Most authors follow a rule of ≥ 40 cycles but it has been argued that for asymptomatic EBOV convalescents, the true LoD may be ≥ 36 cycles. [19] Presence/absence data in other articles [17,21,23,27,30,31,33,35,37] are not shown in Fig 2 but can be used to observe that overall, only 7 of the 145 individually identified patients in our extracted data had detectable virus in blood after day 16 of illness (using LoD ≤ 40). The latest positivity was day 29 of illness, using the criterion that CT value ≤ 40. With a revised CT value detection threshold of 36, the latest date for virus detected in blood among patients in our extracted data would be day 20 [18].

### Other body fluids

**Fig 3:**
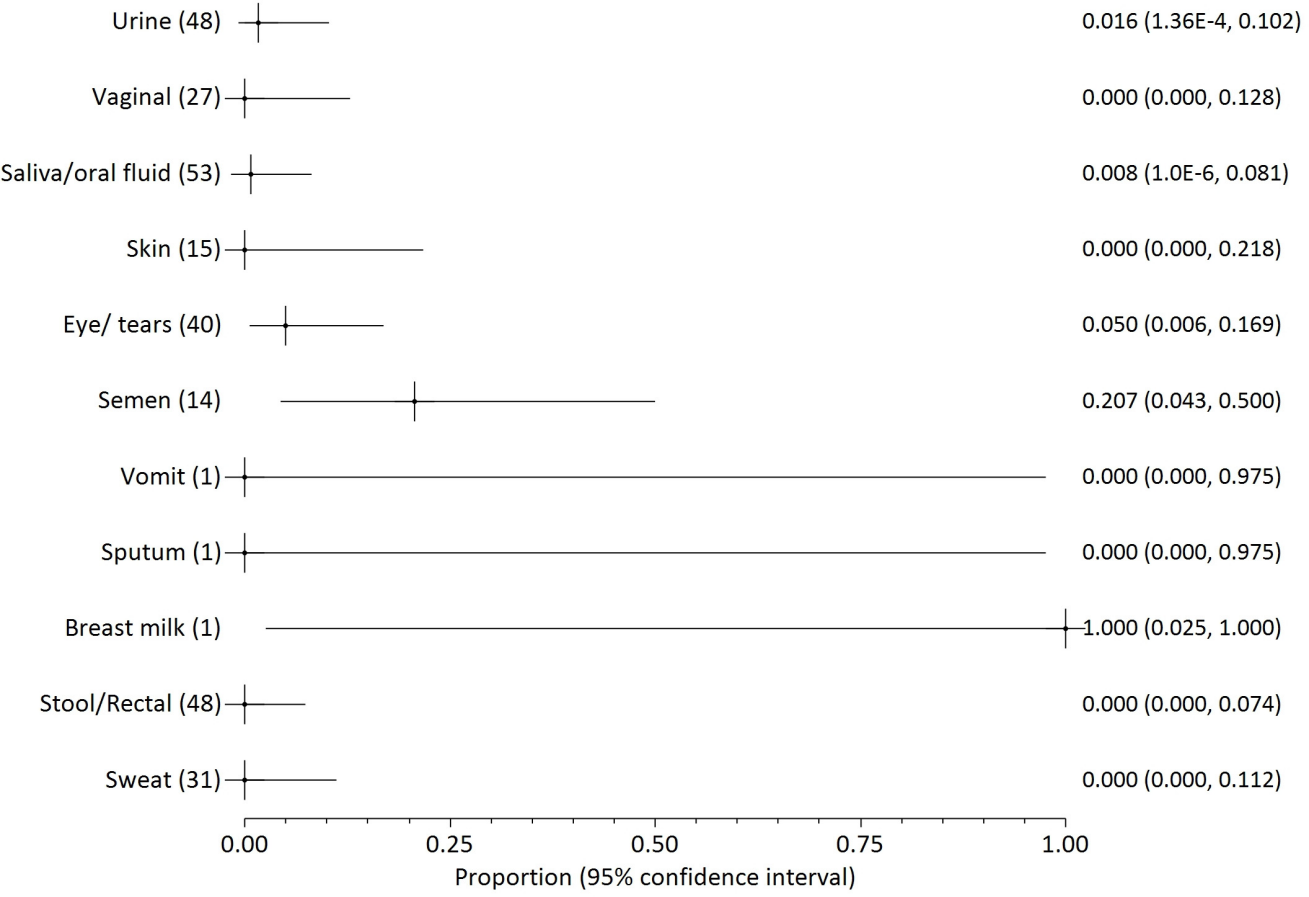
Probability of positivity for all samples tested by culture through day 110 post illness onset. The numbers in parenthesis after each fluid type indicate the number of patients who provided samples for each body fluid. The numbers on the right side are the mean probability for positivity (also shown as a cross mark) with 95% confidence interval in parentheses (shown as lines both sides of the cross).

**Fig 4:**
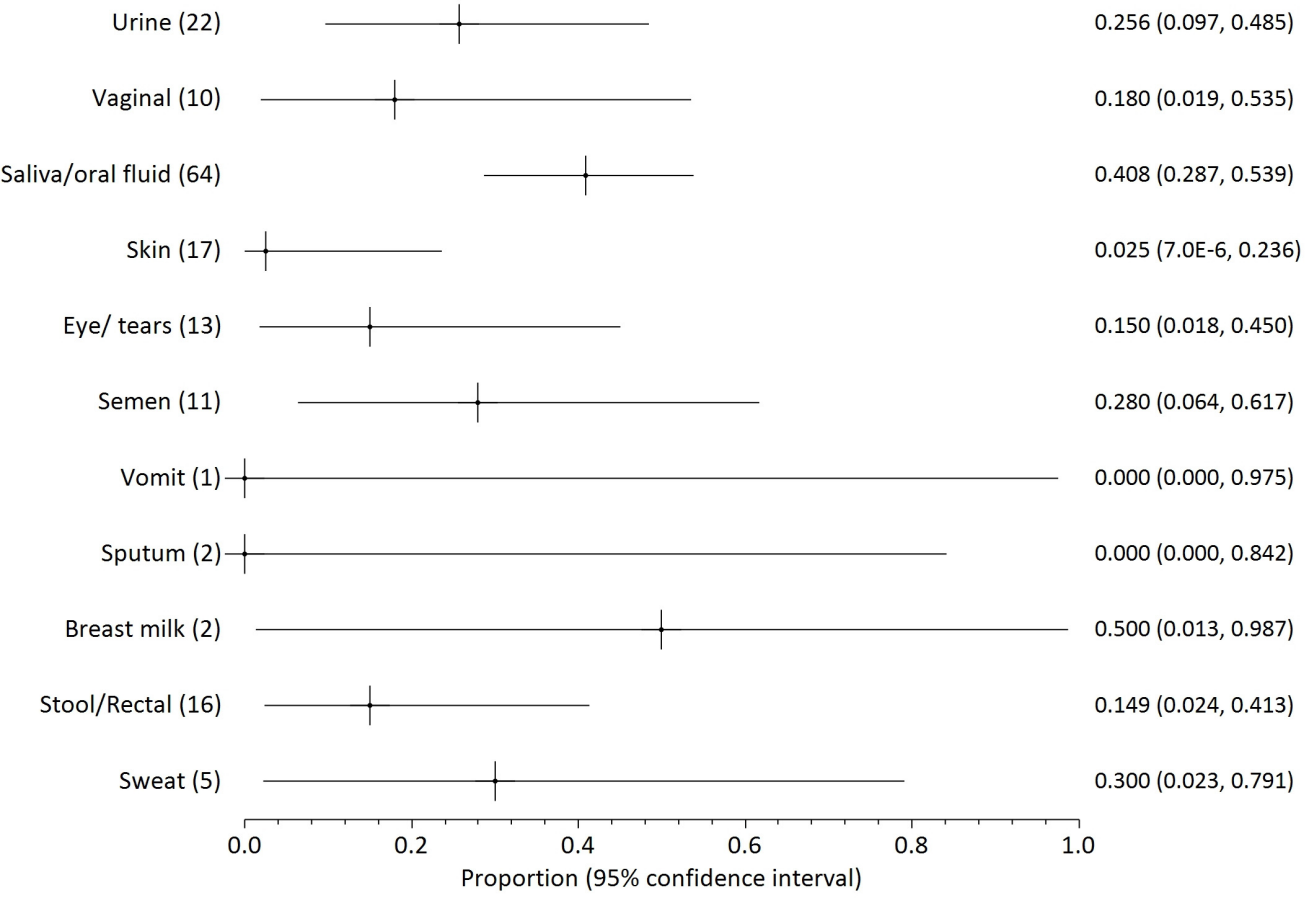
Probability of positivity for all samples tested by RT-PCR through day 110 of illness onset. The numbers in parenthesis after each fluid type indicate the number of patients who provided samples for each body fluid. The numbers on the right side are the mean probability for positivity (also shown as a cross mark) with 95% confidence interval in parentheses (shown as lines both sides of the cross).

**Fig 5:**
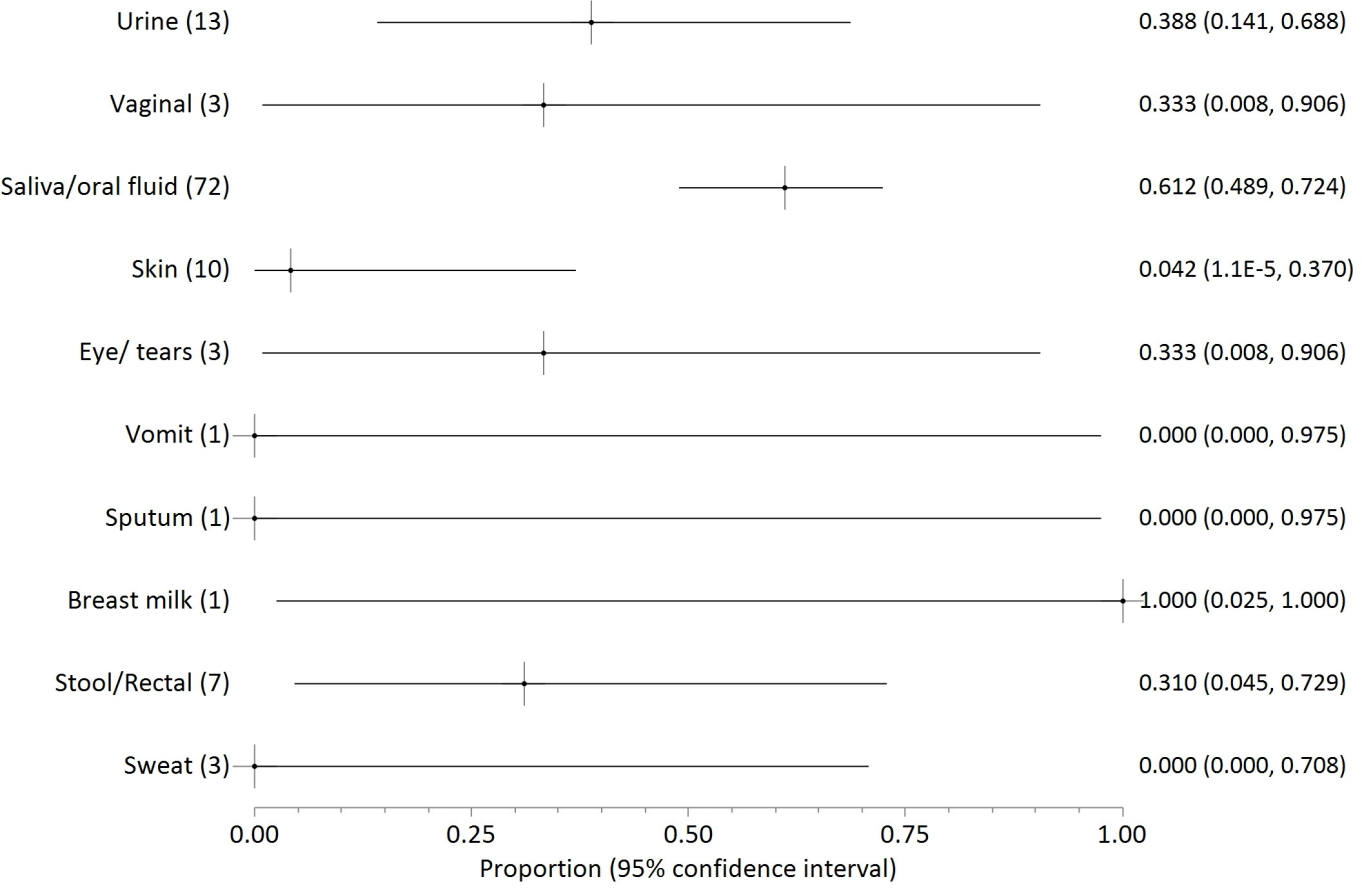
Probability of positivity for all samples tested by RT-PCR days 1–16 of illness onset. The numbers in parenthesis after each fluid type indicate the number of patients who provided samples for each body fluid. The numbers on the right side are the mean probability for positivity (also shown as a cross mark) with 95% confidence interval in parentheses (shown as lines both sides of the cross).

**Fig 6:**
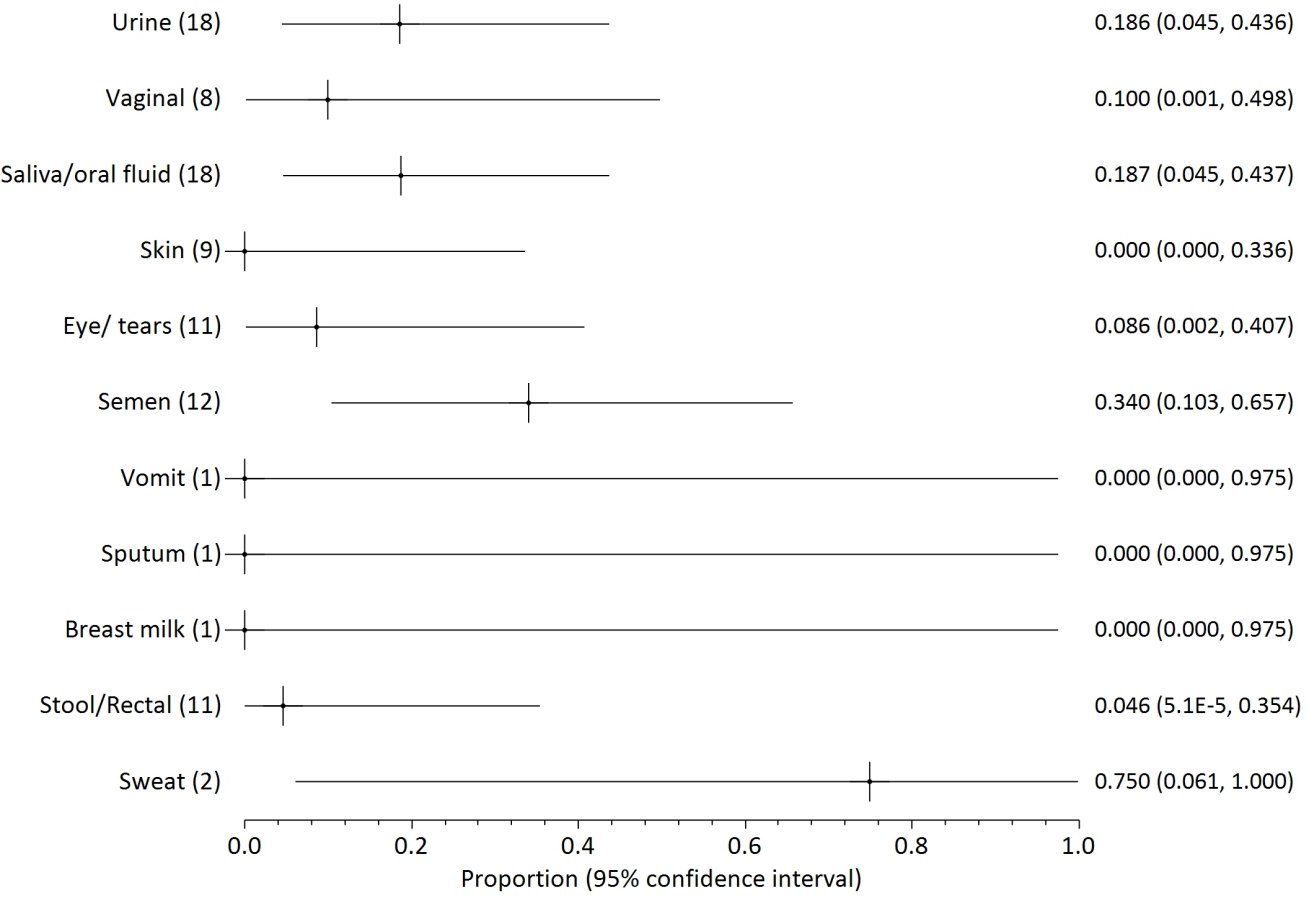
Probability of positivity for all samples tested by RT-PCR days 17–110 of illness onset. The numbers in parenthesis after each fluid type indicate the number of patients who provided samples for each body fluid. The numbers on the right side are the mean probability for positivity (also shown as a cross mark) with 95% confidence interval in parentheses (shown as lines both sides of the cross).

Twenty two papers reported on the presence of filovirus in non-blood body fluids, three of which concerned Marburg virus [41–43], one SUDV [13] and the remainder Zaire Ebola. The proportions of positive samples are illustrated in Figs 3–6, grouped by testing method or date (before day 113 after onset of illness). The numbers in parenthesis after each fluid type indicate the number of patients who provided samples for each body fluid. The numbers on the right side are the mean probability for positivity (also shown as a cross mark) with 95% confidence interval in parentheses (shown as lines both sides of the cross).

We distinguish between results from culture (Fig 3) or by RT-PCR (Fig 4). RT-PCR results are further divided into all early samples (Fig 5, <17 days after onset of illness) or all late samples (Fig 6, >16 days after onset of illness). Days 16/17 are an imperfectly identified typical transition point from active illness to convalescence, because our included papers often did not identify the last date of viraemia or active illness. We observe that blood samples are rarely positive on days 17–112 (Fig 2; virus was detected in blood of just 7 of 145 individuals who can be individually identified in our extracted data). Most deaths from Ebola virus disease occur by day 16 of illness [47,48]; the latest individually identified death in our dataset was on day 16 of illness [23].

Overall, body fluid samples tested by RT-PCR (Fig 4) are about four times more likely to test positive than those tested by culture (Fig 3). This approximate discrepancy also exists among the six studies that provided duplicated results by both culture and RT-PCR [13,16,17,25,30,40]. Results for saliva and other oral fluids are particularly in disagreement (less than 5% positive by culture and almost 50% positive by RT-PCR). Virus was detected in most breastmilk samples, but the dataset is too small (three samples from two patients) to be conclusive. Data on seminal fluid are the most consistent between assay methods, with a mean weighted probability of 21% positivity for culture before day 113, and 28% for RT-PCR. Virus was detected in 73% of seminal fluid samples from 26 samples provided by 18 survivors before day 113. The latest positive result (in seminal fluid) was 203 days after onset of illness, but no results were available for days 111 to 202 or days 204 to 696 after onset of disease. Filovirus was not detected in seminal fluid of six survivors at 697–707 days post-illness [16,17].

Probability of detection by RT-PCR in different body fluids is shown before day 17 (Fig 5) and after day 16 (Fig 6). The small number of patients providing samples leads to large confidence intervals for most body fluids. Saliva is unusual in being provided from over 50 patients in both the early and late monitoring periods which leads to smaller confidence intervals. The probability of virus detection in saliva was fairly similar in both periods, falling only modestly from 55% to 45% before and after 16 days of illness.

Specific viral load data were usually not provided for non-blood samples. From the limited available viral load data, it seems that viral loads can be high or may be almost negligible in non-blood body fluids (high viral load is much more likely to be detected in blood). For instance, very low viral loads were reported in urine (CT values = 36–40+) even when patients had high viraemia [28,29]. For stools or rectal swabs from symptomatic patients, virus was not detected in Kreuels *et al* [25] but viral load in stools was relatively high in two other patients (maximum log 10^5^–10^5.5^ copies/ml: [26,38]). High viral loads for saliva were found in some studies (on days 1–8, lowest CT value = 21, maximum log 10^5.5^–10^6.3^ copies/ml: [15,34,38]), but virus was undetected in saliva of other patients on days 9–16 of illness [17,20,31,38].

## Discussion

We report the first systematic review to investigate the probability of different body fluids being positive for African filoviruses. We summarize evidence about presence and persistence [49] of infectious virus in each body fluid compared to point of onset of illness, which is especially important information when managing disease transmission risks during a large outbreak where there may be many survivors. We provide quantitative estimates of the probability of positivity. Our main finding is that blood from infected patients is likely to be positive for the virus but rarely later than 16 days after the onset of illness. Titres in blood can very high,—up to about 10^8^ viruses/ml; it seems that survival is related to the maximum virus titre [45,50–53]. Many other body fluids are much less likely to be positive, particularly when tested by tissue culture. Although viral loads can be high in some body fluids, virus may be completely undetected, even during active illness. An exception is semen samples, which tended to consistently test positive regardless of whether assay method was RT-PCR or culture.

Infected blood continues to appear to be the most infectious body fluid because it is the body fluid that most consistently tests positive during illness, and viral loads are often observed to be very high. High viral loads are less consistently reported in other bodily fluids. Disease following contact with infected blood is well-documented [7,11,20,54]. Disease transmission from contact with non-blood product body fluids has not been described as clearly, and may depend to some extent on whether the fluid is contaminated with blood (visible or not). Given the known propensity of patients to haemorrhage in later disease, contamination is a plausible explanation. It is noteworthy that patients have been released from hospital when asymptomatic (especially without viraemia) in spite of detected virus in saliva, sweat or urine [25,38] because it was not possible to cultivate virus swabbed from these areas in tissue culture. These results may therefore indicate that recalibration of the limit of detection in at least some filovirus RT-PCR tests in some contexts is advisable (as argued by Spengler et al [19]). It also merits mention that testing usually discontinues on individuals who have already had repeat negative results. Therefore, what Figs 3–6 show is not the likelihood of positivity among all patients or survivors, but rather among those cases for whom positivity is still suspected.

It is also not clear how RT-PCR positivity relates to infectiousness in the absence of a positive culture. Viral RNA can be detected for some time after virus is inactivated [55]. Six of our included studies [13,16,17,25,30,40] suggest that perhaps only about 25% of RT-PCR filovirus-positive samples are infectious.

Two case histories are relevant to human milk exposure, from mother-infant pairs [33]. Both infants were under six months old. Although one mother tested negative in blood and breastmilk for Ebola virus disease (EVD), she was highly symptomatic and her urine tested positive for viral RNA as did her baby (blood test) who subsequently died of EVD, having had 13 days of (not exclusive) breastfeeding during his mother’s active illness. A second breastfeeding mother produced a positive test (blood) for Ebola and highly symptomatic, but her infant (exclusively breastfed for first six days of mother’s illness) did not develop EVD. It is possible that breastmilk alone from mothers with EVD poses low disease transmission risks to unweaned babies. The immunological components in human milk are complex and can reduce transmission of viral diseases from the mother [56]. The chances of EVD transmission from breastmilk alone may be similar to the very low risks of HIV transmission to exclusively breastfed babies [57], risks that rise sharply after the infant starts solids. However, a key difference between HIV and EVD transmission is that close physical contact with the ill mother may mean exposure to other highly infectious bodily fluids from EVD, especially during the most severe stages of illness. The authors of the mother-infant pair case histories highlighted the possible inconsistencies and importance of multiple tests in clinical environments to confirm absence of filovirus infection.

World Health Organisation advice is now that male survivors should adopt barrier methods during sexual contact for at least six months following end of illness [58]. Some accounts [40,59] suggest that six months may still be inadequate; prospective studies [60,61] are under way to gather better evidence about residual virus in seminal products. An epidemiological account by Christie *et al* [39] describes disease transmission to just one of two sexual partners of an EVD survivor; this suggests that viral load in semen may tend to be low or sporadic (low virus load in semen of an EBOV survivor was also reported in Emond *et al* [20]).

One study [34] reported on viral shedding before illness and it seems likely that many body fluids are infectious during at least late incubation. In several cases where virus shedding during incubation was suspected [7,33,62] or shown [34], the incubating patient was either heavily pregnant or in childbirth. It is suspected that immuno-suppression associated with pregnancy can lead to delayed disease onset. Otherwise, data about possible disease transmission from asymptomatic persons are inconclusive [9,16]. There is widespread filovirus antibody seroprevalence in central Africa (2–15% of the population) without history of relevant illness [7–11] suggesting exposure mechanism(s) that can lead to infection without disease. The implications of potentially widespread asymptomatic infection, or the mechanism that lead to it, are unknown. Better understanding of the consequences of low dose exposure that presumably leads to asymptomatic infection, might help to improve disease resistance or to identify optimal intervention points when targeting resources during disease outbreaks. Infection without disease is intriguing because it is widely believed that EVD can be caused by very minimal exposure [63]. EVD resulted in non-human primates which were inoculated with as few as four virions [64], and from aerosol exposure to PFU (log10) 2.6 concentrations of organisms [65]. But it is unclear what the threshold for filovirus infectious dose is in actual community outbreaks among humans [64,66].

Some of the most recent studies in our review discord with older research but also agree with established guidance that was only weakly supported by older studies. For instance, although official guidance has long stated [67] that filovirus was in sweat and any other body fluid, until recently viral RNA was never reported in sweat [11], it still hasn’t been reported in vomit, and is often not found in many other body fluids. Inconsistent test results between selected studies can perhaps also be interpreted with respect to our assessment of validity and study quality. All selected studies used appropriate and clearly described testing techniques, and nearly all used confirmation strategies (multiple tests, different types of tests or laboratories on duplicate specimens and/or healthy controls) to verify results. Most samples were tested relatively quickly (within two weeks), although there was lack of clarity about the test timescale in some papers. The only recurring concern about data collection is that many authors in African environments reported challenges [13,16,17,23,45] in preserving specimen quality (especially preventing breaks in the cold storage chain). These problems may have resulted in some unreliable results [33]. Within our review, virus in non-blood products was often detected by RT-PCR among patients treated in high resource (western) hospital environments [19,25,26,29–31,40]. However, even when RT-PCR suggested positivity, detection by culture in duplicated samples within this group was repeatedly not possible, which was interpreted by clinicians and authors as a very low risk of actual infectivity from these samples of non-blood body fluids [19,25,30]. New data emerging from the most recent outbreaks should adhere to more rigorous storage and testing procedures and thus further clarify the likelihood of infectious virus in specific body fluids.

### Limitations

It is very hard for any systematic review to be fully up-to-date so soon after a large outbreak. Our search only covers research published on and before 23 July 2015. It is not desirable, however, to wait until all data from the 2014–2015 epidemic are available because the scientific information may be needed soon to help manage the next outbreak.

There were biases in how body fluid data were collected. Surveys exist among convalescents regardless of severity of original disease or possible sequelae [16,17], but many data collected after day 16 were on patients who were not representative of most patients in a filovirus outbreak. Many samples after day 16 came from a small number of patients treated in high resource (western) hospitals; these samples were more likely to have virus detected than samples from patients in Africa. It may be reasonable to assume samples collected in in an outbreak area were less reliably stored or processed, and therefore more likely to incorrectly find no virus detected. However, data were also more likely to be collected from patients still exhibiting symptoms (treated at any location), especially viraemia, whereas all testing stopped on patients who recovered quickly. Thus, test results tend to only be available for those who recently tested positive. This bias in patient sample recruitment falsely elevates the apparent probability of late positivity seen in our results. Further difficulties with the reliability of the detection tests include the ever present possibility of false positives by any method, the contradictory results reported from different test assay methods on identical samples (especially culture vs. RT-PCR), or indeed the uncertainty about the appropriate threshold for correct CT values to use when declaring Limit of Detection [19].

### Conclusions

Filovirus has been found in most types of body fluid, but not every sample from every symptomatic patient. Furthermore, with the exception of semen, most non-blood, RT-PCR positive samples are likely to be culture negative and so of low infectious risk. Nevertheless, it is not apparent how relatively infectious many body fluids are, even when culture positive, not least because viral loads are uncertain. Contact with blood and blood-stained body fluids remains the major risk for disease transmission.

## Supporting Information

S1 Table Grey literature sources and selections. (DOCX)

S2 Table PRISMA checklist. (DOCX)

S3 Table Study quality and validity assessment. (DOCX)
