# Supplementary material for: Presence and Persistence of Ebola or Marburg Virus in Patients and Survivors: A Rapid Systematic Review
Source: PLoS Negl Trop Dis. 2016 Feb 29;10(2):e0004475. doi: 10.1371/journal.pntd.0004475 (PMC4771830; doi:10.1371/journal.pntd.0004475)
Supplement: S1 Table — (DOCX) [file pntd.0004475.s001.docx]

Supporting Information for

“Presence and persistence of Ebola or Marburg virus in patients and survivors: A Rapid Systematic Review”

Julii Brainard^a^, Katherine Pond^b^, Kelly Edmunds^a^, Lee Hooper^a^, Paul Hunter^a*^

^a^ University of East Anglia, Norwich NR4 7TJ, UK

^b^ University of Surrey, Guildford GU2 7XH, UK

* Correspondence to = paul.hunter@uea.ac.uk

**Table S1. Grey literature sources and selections**

Notes: All hits were found by searching given source with the terms (*filovir* OR ebola OR ebolavir* OR Marburg-virus*), with further specificity for the WHO website as noted.

| **Source** | **Hits** | **Included** |
| --- | --- | --- |
| www.cdc.gov | 934 | 1 (Martini and Schmidt 1968 [1]) |
| www.opengrey.eu | 47 | 0 |
| www.apa.org | 38 | 0 |
| www.ovid.com Healthstar | 6 | 0 |
| www.docuticker.com | 8 | 0 |
| University of York DARE | 3 | 0 |
| Greylit.org | 62 | 0 |
| www.ntis.org | 7 | 0 |
| www.who.int (screened first 20 results for each body fluid only) , precise search = (Ebola or filovirus or “Marburg virus” ) AND (“viral load” or survival or persistence or disinfection or semen or saliva or vomit or vaginal or rectal or breastmilk or urine or stools or faeces or blood) | 800 (including duplicates) | 0 |

Reference:

1. Martini G, Schmidt H (1968) Spermatogene Übertragung des "Virus Marburg" (Spermatogenesis Transmission of Marburg Virus). Klinische Wochenschrift 46: 398-400.
