# Supplementary material for: Presence and Persistence of Ebola or Marburg Virus in Patients and Survivors: A Rapid Systematic Review
Source: PLoS Negl Trop Dis. 2016 Feb 29;10(2):e0004475. doi: 10.1371/journal.pntd.0004475 (PMC4771830; doi:10.1371/journal.pntd.0004475)
Supplement: S3 Table — (DOCX) [file pntd.0004475.s003.docx]

Supporting Information for

“Presence and persistence of Ebola or Marburg virus in patients and survivors: A Rapid Systematic Review”

Julii Brainard^a^, Katherine Pond^b^, Kelly Edmunds^a^, Lee Hooper^a^, Paul Hunter^a*^

^a^ University of East Anglia, Norwich NR4 7TJ, UK

^b^ University of Surrey, Guildford GU2 7XH, UK

* Correspondence to = paul.hunter@uea.ac.uk

**Table S3. Study Quality and Validity Assessment**

| **Filovirus species** | **Outbreak dates, place and authors** | **Body fluids tested**  **(No. patients : No. samples) Does No. of samples seem adequate for authors’ conclusions?** | **Assay method (s) and case identification: Clearly described what method, who did it and where?** | **Are the assay methods valid?** | **Did the authors attempt to test controls or duplicate samples (at different labs or using different methods)?** | **Was the timescale from collection to testing acceptable (ideally < 2 weeks)?** | **Were the samples collected and stored properly? (storage, pre-sterilisation)** |
| --- | --- | --- | --- | --- | --- | --- | --- |
|  |  |  |  |  |  |  |  |
| MARV | Sept. 1967, Marburg, Martini and Schmidt [1] | Yes, Semen (1:1) | Yes, Presence of virus antigen, wife contracting disease after sexual intercourse and cell culture test | Yes | Yes | Yes | Unclear, but probably yes |
| MARV | Sept. 1967, Marburg, Martini [2] | Yes, Blood (17:17), Stools (5:5), Throat (6:6), Urine (4:4) | Unclear here but documented elsewhere, cell culture virus isolation | Yes | Unclear, may be described well elsewhere | Unclear, but probably yes | Unclear but probably yes |
| MARV | Feb 1975, Johannesburg SA. Gear et al [3] | Yes. Fluid aspirated from eye (1:2), Blood (3:unclear number); other fluids sampled but results not specified | Yes, Vero cell culture, (locations and researchers named) | Yes | Yes, same sample to multiple labs | Yes, gives dates | Unclear (airlifted, “special containers”) |
| SUDV | 2000,  Gulu Uganda. Bausch et al [4] | Yes. Vomit (1-2:2),  Blood (nasal, 1:1), Breastmilk (1:2), Saliva (10:16), Semen (1:2), Sputum (1:2), Stools (4:4), Sweat (1:1), Tears (1:1), Urine (5:11) | Yes, RT-PCR and virus culture, clear what and where/who, although not always clear exactly which specimen went where or when tested. This may be small subset of pts in Sanchez et al 04, not easy to confirm. | Yes | Yes, but they say discrepancies in results. Confusingly reports 2 samples of breastmilk tested +, on days 7, 15 *and* 40 (40 presumed to be an error, implies semen from same pt) | Unclear timescale for tests in Gulu or elsewhere | Yes, although authors still think might have been cold storage problems |
| SUDV | 2000-2001, Uganda . Towner et al [5] | Yes, (many pts but only six in detail, > 1000 samples), Towner et al patients not same fluids as Bausch et al [4] | Yes, labs in local hospitals. RT-PCR (variants), antigen-capture diagnostic assay, plaque assays (Vero cell cultures) | Yes | Yes, comparing sensitivity & reliability of different techniques | Yes, mostly next day | Yes although may not have been perfect |
| SUDV | 2000-2001 Gulu, Uganda  McElroy et al [6] | Yes  May overlap with prev. reports. Blood (94: 135) | Yes, RT-PCR and other immune response markers; minority of pts not lab-confirmed | Yes | No, not truly, although Controlled by culturing in tissue of healthy persons | No, probably 10+ yrs old | Unclear, insufficient info about handling before arrival to lab |
| EBOV | Sep-Oct 1976, Burke et al [7] | Unclear, single pt in Kinshasa. Blood viraemia (1:10) | Yes, Platelet counts as well as antibody titres | Yes (results for this pt, we don’t report on those confirmed by antibody only) | Yes, Controls and All tests repeated at two laboratories with excellent agreement. | Unclear | Unclear, described for some types of samples but not clear for all |
| EBOV | November 1976, Emond et al [8] | Yes, 1 patient only  Blood: 15 samples  Faeces:2  Seminal fluid: 4  Throat swab: 2  Urine: 5 | Yes, Cell cultures and microscope examination | Yes | Unclear, not a consistent programme to check results, they knew the animal had infected him, though | Yes, or they wouldn’t have released him when they did | Unclear (not described) |
| EBOV | Yambio, Sudan 1979  Baron et al [9] | Yes, 189+ specimens tested: 10 demo cases. | Yes, Vero cells and cavy tissue cultures (virus isolation), Sera antibody and isolation of virus in post-mortem tissues | Yes | Yes, healthy controls | Unclear | Yes, but description is very brief |
| EBOV, | Kikwit, 1995. Ksiazek et al [10] | Yes (main discussion is not our question). Unclear number of samples, min. 20, max. 465, all blood products. | Yes,  Vero cell cultures confirmed by IGm and ELISA, exact tests described and tested at CDC. Ksiazek is co-author on most/all the other Kikwit papers, so his techniques are probably replicated throughout this group. Implies most samples did not quantitate well, so problems. | Yes, but not without problems. , good discussion of uncertainties and reliability | Yes, some control/comparator samples | Yes, quick arrival at CDC | Yes, many details |
| EBOV, | Kikwit, 1995. Rodriguez et al [11] | Yes. All usual emitted fluids save breastmilk, 12 pts:1-4 samples each during convalescence; may overlap with others | Yes, acid-guanidine-phenol extraction, -> RT PCR, cell cultures and genetic sequencing. Location and researchers named. | Yes, also detailed in Sanchez et al 99 | Yes, clones tested, and eg: Duplicates in nose swabs, inconsistencies found, attributed to low level of viral RNA present | Unclear | Yes, Sterilisation, Preservation and cold storage procedures described, breaks in chain still possible comment the authors |
| EBOV | Kikwit, 1995  Rowe et al [12] | Yes. Many fluids, (28+:373), mostly collected after illness; could be very small overlap with Rodrigeuz et al [11] | Yes, some patients were identified from clinical history alone after confirmed outbreak (mixed group in results). Virus isolation, RT-PCR, antigens | Yes | Yes, although confirmation was just ELISA / antigens not necessarily 2 viral RNA methods | Unclear | Unclear, insufficient info but describes storage problems that led to discard of one set of specimens |
| EBOV | 1996, South Africa, Richards et al [13] | Blood (1:2)  Semen (1:1) | Yes, culture for blood.  Unclear re semen of index pt who had antibodies & clinical history to confirm from blood sample | Unclear | No | Unclear | Unclear |
| EBOV | Early 2003, Kéllé, Cuvette Ouest, Congo  Formenty et al [14] | Yes, oral and blood specimens from 24 suspected cases & 10 controls | Yes, RT-PCR, antigens and antibodies, genetic sequencing | Yes | Yes, 10 volunteers gave control samples | Unclear or maybe yes. Arrived at lab within 5 days, not clear when tested after that. | Yes, hopefully. But liquid disappeared from vials |
| EBOV | July-Aug 2014, Guinea, Strecker et al [15] | Yes,  Blood (35:38) | Yes, RT-PCR | Yes | Yes (capillary sample comparison & different test) | Yes | Yes |
| EBOV | 2014 Sierra Leone, Liu et al [16] | Yes, Blood (unclear: 266)  Oral (unclear: 49) | Yes, RT-PCR | Yes | No | Unclear, quite possible the delay was large | Unclear |
| EBOV | 2014 Spain, Mora-Rillo et al [17] | Yes,  Blood (1:2).  10 or 12 samples for each of  Eye fluid / Rectal / Saliva / Stool / Sweat / Urine / Vaginal | Yes, RT-PCR and cell culture | Yes | Partly Yes, culture on later days | At least partly Yes (Blood) | Unclear |
| EBOV | Texas 2014, Liddell et al [18] | Yes, Blood (3:23)  Rectal (1:1)  Saliva (2:4)  Skin (1:2)  Sweat (1:1)  Urine (2:10)  Vaginal (2:3) | Yes, RT-PCR | Yes | No | At least partly Yes for blood, Unclear for others | Unclear |
| EBOV | 2014 Africa to USA, Lyon et al [19] | Yes,  Blood (2:26)  Urine (2:17) | Yes, RT-PCR | Yes | No | At least partly Yes (blood), unclear re Urine | Unclear |
| EBOV | July-Oct 2014. Spengler et al [20] | Yes, Blood (4:56) | Yes, RT-PCR and cell cultures. | Yes | Yes, duplicate culture and RT-PCR samples | Unclear | Unclear |
| EBOV | July-Oct 2014. McElroy et al [21] | Yes, Blood. (4: 60) | Yes, qRT-PCR. | Yes. | No. | At least partly Yes | Unclear |
| EBOV | Sep-Oct 2014, USA Connor et al [22] | Yes, Urine (1:1) | Yes, RT-PCR | Yes | No | At least partly Yes | Unclear |
| EBOV | 2014, Kraft et al [23] | Yes, Blood (2:5) | Partly, some unclear | Unclear | Unclear or No | At least partly Yes | Unclear |
| EBOV | August 2014, Sierra Leone, transfer to Germany. Kreuels et al [24] | Yes, Sweat (1:19), Urine (1:18), Tears (1:1), Blood plasma (1:12), Stool/saliva/sputum/  conjunctival = (1:1) | Yes, RT-PCR and Vero cell cultures, labs named, | Yes, methods well described | Yes, confirmation same samples sent to different labs & compared | Yes, not perfect description, but probably within 2 weeks if not 2 days | Unclear |
| EBOV | Oct. 2014, Guéckédou, Guinea  Moreau et al [25] | Yes  Urine (1:1)  Blood (4:6)  Breastmilk (1:1) | Yes, RT-PCR on site, variants identified. | Yes | No | Yes (immediate) | Unclear, likely to be Yes because tested quickly with standard kits |
| EBOV | Oct 2014, transfer to USA Florescu et al [26] | Yes,  Blood (1:24) | Yes, RT-PCR | Yes | No | At least partly Yes. | Unclear |
| EBOV | Oct 2014 transfer to Germany. Wolf et al [27] | Yes,  Blood (1:27)  Rectal (1:9)  Urine (1:8) | Yes, RT-PCR | Yes | No | At least partly Yes (blood) | Unclear |
| EBOV | Oct 2014, New York, Yacisin et al [28] | Yes,  Blood (1:2) | Yes, NAAT | Yes | No | Unclear, probably yes | Unclear |
| EBOV | Nov 2014, convalescent in India, Express News [29] | Yes,  Blood (1:2)  Semen (1:1) | Yes, RT-PCR | Yes | Yes | Yes | Unclear |
| EBOV | Nov 2014 transfer to Switzerland, Schibler et al [30] | Yes,  Blood (1:12)  Eye Fluids (1:3)  Rectal (1:8)  Saliva (1:6)  Sweat (1:3)  Urine (1:11) | Yes RT-PCR | Yes | No | At least partly yes (blood) | Unclear |
| EBOV | Nov 2014, Monrovia. Wong et al [31] | Yes,  Blood (2:6) | Yes, RT-PCR. | Yes | No | Yes | Unclear |
| EBOV | Dec 2014, Akerlund et al [32] | Yes, Blood (1:4)  Oral (1:3)  Skin (1:3)  Urine (1:1)  Vaginal (1:1) | Yes, qRT-PCR. | Yes. | No | Yes. | Unclear, likely to be Yes because tested quickly with standard kits |
| EBOV | March 2015, USA convalescent, Varkey et al [33] | Yes,  Blood (1:2)  Eye (1:3)  Semen (1:1)  Urine (1:1) | Yes, RT-PCR and culture | Yes | Yes for eyes and blood, No for others. | Yes | Yes for eyes, unclear for others. |
| EBOV | March 2015, Liberia. Christie et al [34] | Yes, Blood (1:2)  Semen (1:1) | Yes, RT-PCR. | Yes | No | Yes | Unclear |

Notes: RT-PCR = any of many variants of the reverse transcriptase polymerase chain reaction test.

**References**

1. Martini G, Schmidt H (1968) Spermatogene Übertragung des "Virus Marburg" (Spermatogenesis Transmission of Marburg Virus). Klinische Wochenschrift 46: 398-400.

2. Martini G (1973) Marburg virus disease. Postgraduate medical journal 49: 542-546.

3. Gear S, Cassel GA, Gear AJ, Trappler B, Clausen L, et al. (1975) Outbreak of Marburg virus disease in Johannesburg. British Medical Journal 4: 489-493.

4. Bausch DG, Towner JS, Dowell SF, Kaducu F, Lukwiya M, et al. (2007) Assessment of the risk of Ebola virus transmission from bodily fluids and fomites. Journal of Infectious Diseases 196: S142-S147.

5. Towner JS, Rollin PE, Bausch DG, Sanchez A, Crary SM, et al. (2004) Rapid diagnosis of Ebola hemorrhagic fever by reverse transcription-PCR in an outbreak setting and assessment of patient viral load as a predictor of outcome. Journal of virology 78: 4330-4341.

6. McElroy AK, Erickson BR, Flietstra TD, Rollin PE, Nichol ST, et al. (2014) Biomarker correlates of survival in pediatric patients with Ebola virus disease. Emerging infectious diseases 20: 1683.

7. Burke J, Declerq R, Ghysebrechts G, Pattyn S, Piot P, et al. (1978) Ebola hemorragic-fever in Zaire, 1976 - Report of an International Commission. Bulletin of the World Health Organization 56: 271-293.

8. Emond R, Evans B, Bowen E, Lloyd G (1977) A case of Ebola virus infection. British Medical Journal 2: 541-544.

9. Baron RC, McCormick JB, Zubeir OA (1983) Ebola virus disease in southern Sudan: hospital dissemination and intrafamilial spread. Bulletin of the World Health Organization 61: 997.

10. Ksiazek T, Rollin P, Williams A, Bressler D, Martin M, et al. (1999) Clinical virology of Ebola hemorrhagic fever (EHF): virus, virus antigen, and IgG and IgM antibody findings among EHF patients in Kikwit, Democratic Republic of the Congo, 1995. Journal of Infectious Diseases 179: S177-S187.

11. Rodriguez L, De Roo A, Guimard Y, Trappier S, Sanchez A, et al. (1999) Persistence and genetic stability of Ebola virus during the outbreak in Kikwit, Democratic Republic of the Congo, 1995. Journal of Infectious Diseases 179: S170-S176.

12. Rowe AK, Bertolli J, Khan AS, Mukunu R, Muyembe-Tamfum J, et al. (1999) Clinical, virologic, and immunologic follow-up of convalescent Ebola hemorrhagic fever patients and their household contacts, Kikwit, Democratic Republic of the Congo. Journal of Infectious Diseases 179: S28-S35.

13. Richards GA, Murphy S, Jobson R, Mer M, Zinman C, et al. (2000) Unexpected Ebola virus in a tertiary setting: clinical and epidemiologic aspects. Critical care medicine 28: 240-244.

14. Formenty P, Leroy EM, Epelboin A, Libama F, Lenzi M, et al. (2006) Detection of Ebola virus in oral fluid specimens during outbreaks of Ebola virus hemorrhagic fever in the Republic of Congo. Clinical Infectious Diseases 42: 1521-1526.

15. Strecker T, Palyi B, Ellerbrok H, Jonckheere S, de Clerck H, et al. (2015) Field evaluation of capillary blood samples as a collection specimen for the rapid diagnosis of Ebola virus infection during an outbreak emergency. Clinical Infectious Diseases: civ397.

16. Liu L, Sun Y, Kargbo B, Zhang C, Feng H, et al. (2015) Detection of Zaire Ebolavirus by Real-Time Reverse Transcription-Polymerase Chain Reaction, Sierra Leone, 2014. Journal of Virological Methods 222: 62-65.

17. Mora-Rillo M, Arsuaga M, Ramírez-Olivencia G, de la Calle F, Borobia AM, et al. (2015) Acute respiratory distress syndrome after convalescent plasma use: treatment of a patient with Ebola virus disease contracted in Madrid, Spain. Lancet Respiratory Medicine 3: 554-562.

18. Liddell AM, Davey Jr RT, Mehta AK, Varkey JB, Kraft CS, et al. (2015) Characteristics and clinical management of a cluster of 3 patients with Ebola virus disease, including the first domestically acquired cases in the United States. Annals of internal medicine Online from 12 May 2015.

19. Lyon GM, Mehta AK, Varkey JB, Brantly K, Plyler L, et al. (2014) Clinical care of two patients with Ebola virus disease in the United States. New England Journal of Medicine 371: 2402-2409.

20. Spengler JR, McElroy AK, Harmon JR, Ströher U, Nichol ST, et al. (2015) Relationship Between Ebola Virus Real-Time Quantitative Polymerase Chain Reaction–Based Threshold Cycle Value and Virus Isolation From Human Plasma. Journal of Infectious Diseases: jiv187.

21. McElroy AK, Akondy RS, Davis CW, Ellebedy AH, Mehta AK, et al. (2015) Human Ebola virus infection results in substantial immune activation. Proceedings of the National Academy of Sciences of the United States of America 112: 4719-4724.

22. Connor MJ, Kraft C, Mehta AK, Varkey JB, Lyon GM, et al. (2014) Successful delivery of RRT in Ebola virus disease. Journal of the American Society of Nephrology: ASN. 2014111057.

23. Kraft CS, Hewlett AL, Koepsell S, Winkler AM, Kratochvil CJ, et al. (2015) The use of TKM-100802 and convalescent plasma in 2 patients with Ebola virus disease in the United States. Clinical Infectious Diseases: civ334.

24. Kreuels B, Wichmann D, Emmerich P, Schmidt-Chanasit J, de Heer G, et al. (2014) A case of severe Ebola virus infection complicated by gram-negative septicemia. New england journal of medicine 371: 2394-2401.

25. Moreau M, Spencer C, Gozalbes J, Colebunders R, Lefevre A, et al. (2015) Lactating mothers infected with Ebola virus: EBOV RT-PCR of blood only may be insufficient. Euro surveillance 20: 3.

26. Florescu DF, Kalil AC, Hewlett AL, Schuh AJ, Stroher U, et al. (2015) Administration of Brincidofovir and Convalescent Plasma in a Patient with Ebola Virus Disease. Clinical Infectious Diseases: civ395.

27. Wolf T, Kann G, Becker S, Stephan C, Brodt H-R, et al. (2015) Severe Ebola virus disease with vascular leakage and multiorgan failure: treatment of a patient in intensive care. Lancet 385: 1428-1435.

28. Yacisin K, Balter S, Fine A, Weiss D, Ackelsberg J, et al. (2015) Ebola virus disease in a humanitarian aid worker-new york city, october 2014. MMWR: Morbidity and mortality weekly report 64: 321-323.

29. Express News Service (2014) ‘Cured’ of Ebola disease in Liberia, Indian man isolated in Delhi after a positive test The Indian Express.

30. Schibler M, Vetter P, Cherpillod P, Petty TJ, Cordey S, et al. (2015) Clinical features and viral kinetics in a rapidly cured patient with Ebola virus disease: a case report. Lancet Infectious Diseases Available online from 19 July 2015.

31. Wong KK, Perdue CL, Malia J, Kenney JL, Peng S, et al. (2015) Supportive care of the first two Ebola virus disease patients at the Monrovia Medical Unit. Clinical Infectious Diseases: civ420.

32. Akerlund E, Prescott J, Tampellini L (2015) Shedding of Ebola Virus in an Asymptomatic Pregnant Woman. New England Journal of Medicine 372: 2467-2469.

33. Varkey JB, Shantha JG, Crozier I, Kraft CS, Lyon GM, et al. (2015) Persistence of Ebola Virus in Ocular Fluid During Convalescence. New England Journal of Medicine 372: 2423-2427.

34. Christie A, Davies-Wayne GJ, Cordier-Lasalle T, Blackley DJ, Laney AS, et al. (2015) Possible sexual transmission of Ebola virus—Liberia, 2015. MMWR: Morbidity and Mortality Weekly Report 64: 1-3.
